# Fecal Calprotectin and Clinical Disease Activity in Pediatric Ulcerative Colitis

**DOI:** 10.1155/2013/179024

**Published:** 2013-02-26

**Authors:** Kaija-Leena Kolho, Dan Turner

**Affiliations:** ^1^Children's Hospital, Helsinki University Central Hospital, P.O. Box 281, 00029 Helsinki, Finland; ^2^Pediatric Gastroenterology Unit, Shaare Zedek Medical Center, The Hebrew University of Jerusalem, P.O. Box 3235, Jerusalem 91031, Israel

## Abstract

*Objective*. To explore fecal calprotectin levels in pediatric ulcerative colitis (UC) in relation with the validated clinical activity index PUCAI. *Methods*. This study included all 37 children (median age 14 years) with UC who had calprotectin measured (PhiCal ELISA Test) by the time of PUCAI assessment at the Children's Hospital of Helsinki in a total of 62 visits. Calprotectin values <100 **μ**g/g of stool were considered as normal. The best cut-off value of each measure to predict 3-month clinical outcome was derived by maximizing sensitivity and specificity. *Results*. In clinically active disease (PUCAI ≥ 10), calprotectin was elevated in 29/32 patients (91% sensitivity). When in clinical remission, 26% (8/30) of the children had normal calprotectin but 7 (23%) had an exceedingly high level (>1000 **μ**g/g). The best cut-off value for calprotectin for predicting poor outcome was 800 **μ**g/g (sensitivity 73%, specificity 72%; area under the ROC curve being 0.71 (95%CI 0.57–0.85)) and for the PUCAI best cut-off values >10 (sensitivity 62%, specificity 64%; area under the ROC curve 0.714 (95%CI 0.58–0.85)). *Conclusion*. The clinical relevance of somewhat elevated calprotectin during clinical remission in pediatric UC is not known and, until further evidence accumulates, does not indicate therapy escalation.

## 1. Introduction

Neutrophil-derived markers, such as fecal calprotectin or lactoferrin, have proven to correlate well with mucosal inflammation of ulcerative colitis (UC) at a rho range of 0.6–0.8 [1–4]. Fecal level of these markers reflects the mucosal influx of inflammatory cells in the gut. When the level of these markers is low, the presence of active inflammation in the colon is unlikely [3, 5–8]. The probability of 1-year remission in UC children with normal calprotectin may be as high as 75% [9]. On the other hand, in UC (and unlike in Crohn's disease), clinical judgment of symptoms also correlates well with endoscopic appearance, at a range of rho 0.7–0.8 [10], and clinical assessment also predicts well clinical outcomes in both adult [11] and pediatric severe UC [12]. Recently, we showed that calprotectin levels rarely decline completely during therapy with glucocorticoids or TNF-*α*-antagonist agents suggesting ongoing inflammation in the majority [13, 14].

The PUCAI is the validated pediatric ulcerative colitis activity index showing good correlation with endoscopic disease activity [15–17]. The index is based on item scores reflecting the clinical situation within the last two days and may thus be used in the acute setting [15]. In acute severe colitis, PUCAI reflects therapeutic response better than calprotectin, the levels of which do not change as rapidly along improvement as the PUCAI scores [18, 19]. There are, however, limited data on the performance of fecal markers related to simple clinical assessment of pediatric UC in clinical practice. We aimed to explore calprotectin levels in relation to clinical disease activity assessed with the validated activity index PUCAI in pediatric UC.

## 2. Subjects and Methods

This retrospective study included all children with UC who had calprotectin measured and clinical data recorded for the assessment of the PUCAI at the Children's Hospital of Helsinki from December 2005 to January 2012, most samples being from the three recent years. Diagnosis of UC was based on typical clinical presentation as well as upper and lower endoscopy [20].

Clinical disease activity was scored using the PUCAI consisted that of six clinical items scored from 0 to 85 points ( abdominal pain 0–10; rectal bleeding 0–30; stool consistency 0–10; number of stools per 24 hours (0–15); nocturnal stools (0–10), and activity level (0–10)). Cut-off scores for remission (PUCAI < 10 points), mild (10–34), moderate (35–64)) and severe (≥65) disease activity have been validated on three different cohorts with sensitivity and specificity of >90% [10, 15, 16].

Three-month clinical outcome was assessed using a physicians global assessment (PGA) from 0 (remission) to 3 (severe disease). Good outcome was defined as no need to change maintenance medication, successful weaning of steroids when applicable, and disease activity of no more than 1 according to PGA. 

Children provided a stool sample within two days of the time of PUCAI assessment. The samples in children undergoing endoscopy were taken prior to bowel cleansing [21]. Fecal calprotectin was measured using the PhiCal ELISA Test (Nova Tec Immunodiagnostica, Dietzenbach, GmBH, Germany). Calprotectin value <100 *μ*g/g of stool was considered as normal whereas values >1000 *μ*g/g were considered as exceedingly high [9, 13, 14]. 

### 2.1. Ethics

The patients involved in this study participated in a study on pediatric inflammatory bowel disease approved by the Ethics Committee of the Helsinki University Central Hospital. The patients/their guardians signed an informed consent form when entering the study. 

### 2.2. Statistical Analyses

Data are presented as means (±standard deviation) or medians (interquartile range) as appropriate for the distribution normality. Correlations between individual parameters were sought using Spearman correlation. To compare the predictive utility of the PUCAI as compared with calprotectin, area under the receiver operating characteristics (ROC) curve (±95% CI) was used. The best cut-off values of each measure to predict 3-month clinical outcome were derived by maximizing sensitivity and specificity. All analyses were performed using SPSS V16. 

## 3. Results

A total of 62 visits were included from 37 different children with fecal calprotectin values available by the time of the assessment of the PUCAI ( 18 (49%) males, median age 14.3 years (IQR 13.0–15.8, 33 (89%) with extensive disease and the others with left-sided disease; Table 1). Fourteen (38%) children were at disease onset and the others with median disease duration of 1.5 (IQR 0.7–3.0) years. Sixteen of the visits were during clinical remission (judged by PGA), 16 (26%) during mild disease activity, 12 (19%) during moderate disease activity, and 18 (29%) during severe disease activity. Median number of samples per patient was 1 (IQR: 1-2, range 1–5). 

**Figure 1:**
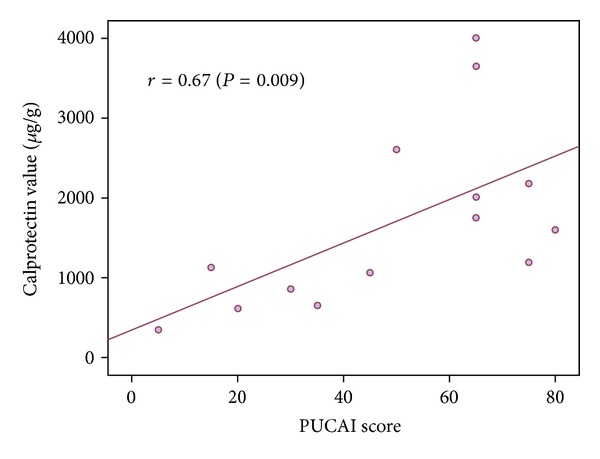
The Spearman correlation of the pediatric ulcerative activity index (PUCAI) and fecal calprotectin in pediatric patients with ulcerative colitis undergoing diagnostic endoscopy.

In the total sample set, the correlation of calprotectin and the PUCAI was good (*r* = 0.53, *P* < 0.001, *n* = 62). The correlation between PUCAI and calprotectin among the 14 children at diagnosis (all with clinically active disease) was higher (*r* = 0.67, *P* = 0.009; Figure 1).

**Figure 2:**
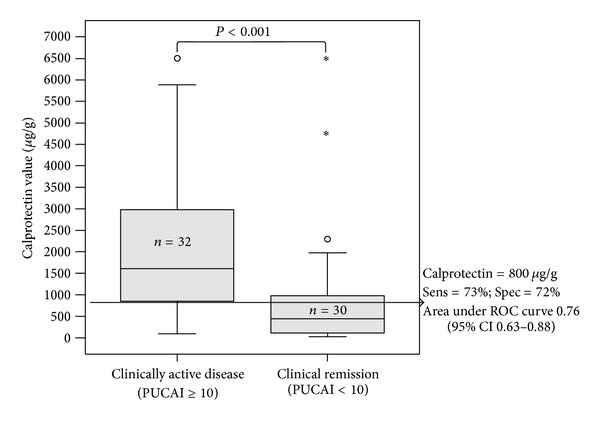
The relation of the pediatric ulcerative activity index (PUCAI) and fecal calprotectin in pediatric patients with ulcerative colitis. The arrow shows the best fecal calprotectin cutoff (by maximizing sensitivity and specificity) to identify patients in clinical remission according the PUCAI.

The best calprotectin cutoff to identify the 30/62 visits in clinical remission was <800 *μ*g/g (sensitivity 73% and specificity 72%; area under the ROC curve of 0.76 (95% CI 0.63–0.88); Figure 2). The values were not different when considering only one observation per patient to avoid possible repeated measures bias (the first visit per patient; *n* = 37) with an area under the ROC curve of 0.71 (0.53–0.89) and similar cut-off value.

Calprotectin was exceedingly high in all 13 samples collected during a severe attack (i.e., PUCAI ≥ 65) (median 2179 *μ*g/g range 1190–9504 (IQR 1682–5310)). All those with PUCAI scores reflecting moderate-severe disease (40–85) had elevated calprotectin values. In mild-to-moderate disease (PUCAI 10–64, *n* = 19) calprotectin was elevated in all except two patients (median 829 *μ*g/g, range 7–7287 (IQR 416–1599)). Calprotectin was completely normal in 8 of 30 assessments performed during clinical remission (26%). In seven (23%), calprotectin level was exceedingly high (>1000 *μ*g/g). In four of these, the assessments were done on the day of maintenance infusion of infliximab, in two cases steroids had been tapered within one-to-two weeks earlier and, one was on only 5-ASA. Three of these seven (two on infliximab, one in whom steroids were tapered off) remained in sustained-steroid-free clinical remission 3-months after the sample date, despite the very high calprotectin level. 

The PUCAI performed just as well as calprotectin in predicting 3-month clinical outcome (poor versus good outcome), area under the ROC curve being 0.71 (95% CI 0.57–0.85) versus 0.714 (95% CI 0.58–0.85), respectively. The best cutoff for calprotectin for predicting poor outcome was 800 *μ*g/g (sensitivity 73%, specificity 72%) and for the PUCAI best cut-off >10 (sensitivity 62%, specificity 64%).

Of the 30 assessments in clinical remission, 20 (66%) remained in steroid-free clinical remission for the forthcoming three months. All 10 assessments of both clinical remission or mild clinical disease (i.e., PUCAI < 35), and normal calprotectin <100, remained in steroid-free remission in 3 months. The calprotectin values and outcome of the patients in remission or having mild-to-moderate disease activity according to the PUCAI during the following three months ara shown in Table 2.

## 4. Discussion

The use of calprotectin as a surrogate marker for intestinal inflammation is emerging. However, the data on calprotectin related to clinical disease activity in children with UC are sparse. We show good correlation between the PUCAI and fecal calprotectin. In the clinically severe disease (PUCAI > 65), calprotectin was exceedingly high (>1000 *μ*g/g) in all cases and did not bring any additional information to clinical assessment. It is worthwhile noting that the PUCAI is much more responsive to a rapid change than calprotectin in severe disease; that is, the PUCAI shows a sharp decrease within only a few days of starting effective medication [18] reflecting the well-known notion that mucosal healing lags after clinical remission. On the other hand, some of those in clinical remission according to PUCAI still had elevated calprotectin levels, but in the majority the levels were only moderately elevated. Our data suggests, however, that closer monitoring should be offered to the patients in clinical remission but with exceedingly high calprotectin level since many of them may show deterioration within the forthcoming weeks. It may also suggest that the current therapies are unable to induce complete attenuation of mucosal inflammation in a large proportion of the pediatric patients. Recently, we showed that only one-third of 41 children with UC had normal calprotectin levels while 13% had exceedingly high levels despite all being in longstanding clinical remission defined using the PGA (i.e., 80% with at least 6-month duration followup) [9]. 

Although mucosal healing has been shown in adults, to predict favourable clinical outcome in UC [11, 22, 23] is yet to be proven that this is superior to clinical assessment of remission. Indeed, in the combined ACT cohorts (466 adults with UC treated with either infliximab or placebo) endoscopic healing after 8 weeks of therapy predicted 1-year colectomy overall but not among the subset of patients with clinical remission [22]. Moreover, clinical judgment of response to steroid treatment in acute severe UC has been shown to predict long-term, clinically important, outcomes, both in adults [11] and in children [12, 24], irrespective of endoscopic healing. Similarly here, we could not find any major difference between clinical (PUCAI) and endoscopic (calprotectin) assessment of disease activity with regards to predicting 3-month steroid free remission although the sensitivity and specificity of calprotectin were somewhat higher than the PUCAI. This should be interpreted with caution given the small sample size, the retrospective design, and the relatively short follow-up duration.

Notably, it remains an unanswered question whether immunosuppressive therapy should be escalated during clinical remission solely based on the presence of elevated calprotectin. The question is not merely if calprotectin levels during remission are independently predictive, but if the predictive power is large enough to justify the associated adverse events. In other words, how many children in remission will be required to step up from 5-ASA to thiopurines or even anti-TNF in order to obtain one favourable outcome (i.e., number needed to treat). Currently, this cannot be recommended until evidence is available to show that this aggressive approach indeed leads to better outcomes.

Furthermore, the target level of fecal surrogate markers to define acceptable therapeutic response is not known. In a previous study, 89% of 25 young patients with Crohn's disease in clinical remission remained in remission for nine months if their fecal calprotectin level was lower than 400 *μ*g/g [25]. Here, the best cutoff for calprotectin for predicting 3-month poor outcome was as high as >800 *μ*g/g and for the PUCAI scores ≥10 (which is the PUCAI definition for active disease) with no major difference in sensitivity and specificity. 

In conclusion, in pediatric UC, clinical disease activity assessed with the validated index PUCAI shows good correlation with the levels of calprotectin. At present, the clinical relevance of elevated calprotectin level when in sustained clinical remission is not known and, until known otherwise, does not indicate escalating therapy based on this isolated finding only. However, it may indicate closer monitoring, especially in those with values >800 *μ*g/g. 

## Figures and Tables

**Table 1:** Background data of the 37 pediatric patients with ulcerative colitis.

Age (years)	14 (median, range 4.5–17)
Gender	Male *n* = 18
Disease duration (years)	1.5 (median, range 0–5)
Disease extension	Pancolitis *n* = 33
	Left-sided colitis *n* = 4
Medication	5-ASA *n* = 13
	5-ASA and/or azathioprine *n* = 5
	Infliximab *n* = 8
	Glucocorticoids *n* = 11
	None *n* = 11

**Table 2:** Three-month outcome of pediatric patients with mild-to-moderate ulcerative colitis according to the clinical disease activity index PUCAI and fecal calprotectin at baseline. The outcome was determined according to physicians global assessment.

No.	Disease activity according to the PUCAI (total score)	Calprotectin *µ*g/g of stool	Outcome within 3 months
1	Remission (<10)	14	Remission
2		14	Remission
3		16	Remission*
4		30	Remission
5		45	Remission
6		69	Remission
7		69	Remission
8		90	Remission
9		131	Remission
10		155	Flare (within two months)
11		189	Flare (when tapering corticoid)
12		333	Remission*
13		345	Flare
14		372	Remission
15		411	Remission
16		441	Remission
17		521	Remission
18		532	Flare (when tapering corticoid)
19		581	Remission
20		628	Remission
21		663	Flare (within a month)
22		796	Remission
23		902	Flare*
24		1052	Remission*
25		1173	Flare*
26		1233	Remission
27		1976	Flare (when tapering corticoid)
28		2295	Flare*
29		4518	Flare (within two weeks)
30		9625	Remission
31	Mild-to-moderate disease (10–64)	7	Remission
32		55	Remission
33		87	Remission
34		327	Flare (when tapering corticoid)
35		416	Remission
36		611	Lost from followup
37		653	Flare
38		751	Flare
39		766	Flare
40		829	Flare (ongoing)
41		856	Remission
42	Mild-to-moderate disease (10–34)	1067	Flare
43		1110	Remission
44		1127	Remission
45		1599	Flare (when tapering corticoid)
46		1667	Flare (within two weeks)
47		1911	Flare (ongoing)
48		2604	Flare
49		7287	Flare (when tapering corticoid)

*Sample taken on the day of infliximab administration.
